# Mastoidectomy in surgical procedures to treat retraction pockets: a single-center experience and review of the literature

**DOI:** 10.1007/s00405-022-07573-7

**Published:** 2022-08-10

**Authors:** Angelo Immordino, Pietro Salvago, Federico Sireci, Francesco Lorusso, Palmira Immordino, Dario Saguto, Francesco Martines, Salvatore Gallina, Francesco Dispenza

**Affiliations:** 1 Unit of Otorhinolaryngology, Department of Biomedicine, Neuroscience and Advanced Diagnostics, Azienda Ospedaliera Universitaria Policlinico ‘‘Paolo Giaccone’’, University of Palermo, Via del Vespro, 133, 90127 Palermo, Italy; 2 Unit of Audiology, Department of Biomedicine, Neuroscience and Advanced Diagnostics, AOUP Paolo Giaccone, University of Palermo, Via del Vespro, 133, 90127 Palermo, Italy; 3 Hygiene and Preventive Medicine Section, Department of Health Promotion, Maternal and Infant Care, Internal Medicine and Medical Specialties, Azienda Ospedaliera Universitaria Policlinico ‘‘Paolo Giaccone’’, University of Palermo, Via del Vespro 129, 90127 Palermo, Italy; 4 Department of Biomedicine, Neuroscience and Advanced Diagnostics, Azienda Ospedaliera Universitaria Policlinico ‘‘Paolo Giaccone’’, University of Palermo, Via del Vespro 133, 90127 Palermo, Italy

**Keywords:** Retraction pocket, Tympanic atelectasis, Tympanoplasty, Mastoidectomy

## Abstract

**Purpose:**

Retraction pocket (RP) is a common event affecting the middle ear when a negative pressure within it causes a retraction of a single part of the tympanic membrane (TM). Patients can be asymptomatic or can experience hearing loss, fullness feeling and/or ear discharge. RP can be stable or develop a cholesteatoma; aim of the study was to investigate if mastoidectomy may play a role in the surgical management of patients suffering from RP, both reporting our experience and discussing the existing literature.

**Methods:**

Fifty-one patients affected by RP were referred for surgery and randomly divided into two groups. Patients of G1 group underwent tympanoplasty with mastoidectomy, patients of G2 group underwent tympanoplasty only. A systematic review of the literature was then carried out by applying the PRISMA guidelines.

**Results:**

The mean follow-up lasted about 36 months. The G1 and G2 groups reached a postoperative mean air–bone gap (ABG) of 7.1 dB HL and 5.1 dB HL, respectively, with a mean ABG improvement of 13.2 dB HL and 12.4 dB HL. An ABG improvement was observed in the 59.7% of the G1 group and in the 63.2% of the G2 group, respectively (*p* > 0.5). Only one case of long-term complication was recognized in the G1 group. We combined, integrated and analyzed results of our prospective study with results of the literature review.

**Conclusions:**

Based on the combined results of our study and literature review we may conclude that there is no evident benefit in performing mastoidectomy for the treatment of RP. In fact, no differences in ABG improvement or in RP recurrence were reported between the two groups.

## Introduction

Tympanic atelectasis is a condition characterized by the displacement of the tympanic membrane (TM) towards the structures of the middle ear (ME). This condition is determined by the progressive development of a negative pressure at the level of the ME. The retraction can affect the entire TM or a single part of it, in the latter case this condition is called retraction pocket (RP) and can develop at the level of the pars tensa or pars flaccida. The retraction, during its progression, can first lead to contact with the ossicular chain, causing erosion of the incudo-stapedial joint, in its most advanced forms it can lead to contact with the promontory and its extension towards the epitympanum, the hypotympanum and the eustachian tube.

Eustachian tube dysfunction (ETD) and a progressive thinning of the tympanic membrane are frequent concurrent causes of RP as well as recurrent episodes of otitis media with effusion [1]. By the time the patient is visited, ETD may no longer be a risk factor, leaving a tympanic membrane segment which remains retracted primarily due to lack of stiffness and stretching of tympanic membrane layers which occurred during the development of the pocket. The most common site of RP is the posterior–superior quadrant of the pars tensa, which is also the most diagnosed syndrome in symptomatic patients.

Patients can be unaware of RP for long time until they experience hearing loss, fullness feeling and/or ear discharge. RP might cause a mild to moderate conductive hearing loss which affects mainly low to middle frequencies.

From a prognostic point of view, RP can be stable and self-cleansing or develop a frank cholesteatoma; Cassano et al. reported a 20% of progression from a simple RP to a cholesteatoma, while Kasbekar et al. found a cholesteatoma in the 31% of cases of retracted tympanic membrane [2, 3]. For this reason, RP requires a follow-up to early identify any complication and address the patient to surgical treatment.

Sadè was the first to classify the RP into four degree of severity: I, mild retraction; II, retraction with contact to incudo-stapedial joint; III, TM retraction without adhesion to the promontory; IV, TM in contact with the promontory [4, 5]. Through pneumatic otoscopy, it is possible to demonstrate the absence of movements of the tympanic membrane of the last degree.

The treatment of RP is highly variable amongst otologists and nowadays there is no unanimous agreement regarding the indication and the methods of treatment [6].

The basis of this lack of consensus is the impossibility of predicting which RPs will undergo evolution into cholesteatoma and which will remain stable and asymptomatic [7].

There are different therapeutic options for this condition, these include conservative or surgical strategies, such as ventilation tube insertion, RP excision with TM reconstruction and canal wall-up or canal wall-down tympanoplasty [8].

The main purpose of the present article is to investigate whether mastoidectomy may play a role in the surgical management of patients suffering from RP, both reporting our experience and discussing the results of a review of existing literature.

## Materials and methods

### Study sample

We conducted a prospective study enrolling 51 patients who were affected by RP and were referred at the ENT Department for surgery.

Before audiological examination, all participants underwent a detailed collection of the anamnesis (including personal information, onset of the symptoms, hearing complaints and any previous medical and/or surgical treatment performed) and a complete otolaryngologic examination with micro-otoscopy and endoscopy of the upper airways.

Audiometric tests and Eustachian tube function tests were performed in all subjects studied.

Hearing loss severity was classified preoperatively by evaluating the pure-tone audiometry (PTA) at 0.5–1–2–4 kHz and the difference between air and bone conduction was calculated both preoperatively and postoperatively as air–bone gap (ABG).

Patients were included in our study if they met the following criteria: grades III or IV of TM retraction, peripheral RP, RP with not visible fundus, non-self-cleaning RP and recurrent ear discharge. Patients affected by cholesteatoma were excluded from the study. All subjects included underwent a preoperative temporal bone computed tomography (CT) [9, 10].

The study protocol was then exposed to the patients who, after a complete counceling, gave their written consent. The approval to carry out this study was also obtained from the ethics committee of our hospital (Ref. ORL-05/011).

The recruited patients were then referred randomly to two different surgical procedures: patients of G1 group underwent to tympanoplasty with canal wall-up mastoidectomy, patients of G2 group underwent to tympanoplasty only. If necessary, ossiculoplasty was also performed. The surgical procedures were all performed by the same expert surgeon. The randomization of patients in the two groups was carried out using the specific function on "R" software. During the first post-operative month, patients underwent weekly follow-ups to perform dressings and check the status of the grafts. Long-term follow-up was carried out for at least 12 months. Primary and secondary outcome were evaluated during long-term follow-up: non-recurrence of the RP was considered as the primary outcome; the improvement of the air conduction threshold with ABG reduction was instead considered as a secondary outcome. The data clusters were expressed as a percentage, while the quantitative variables were expressed as mean ± standard deviations. The statistical processing of the data was performed by applying the Chi-squared test, Fischer test and/or Student's *t* test using R-software (R-software inc. 2 Shaw Alley, San Francisco CA 94105).

### Surgical procedure

All the patients were treated under general anesthesia and approached with a postauricular incision to access the ME. Ipsilateral fascia graft was harvested during the approach to the TM.

In all cases RP was excised and the TM defect was reconstructed using temporalis muscle fascia graft placed with an underlay technique. The fascia graft was reinforced using tragal cartilage in all patients in whom ETD was detected on preoperative Eustachian tube function tests. Furthermore, in our case series, these group of patients also presented bone erosion at the scutum level. The cartilage graft was harvested with its perichondrium by making an incision along the tragal free edge, then it was placed medial to the fascia graft. When ossicular erosion was detected an ossiculoplasty with autologous incus was performed if it was present and free from pathological changes, in the remaining cases it was preferred to perform it with a titanium prosthesis. In addition to the procedures previously described a canal wall-up mastoidectomy was performed in G1 group cases.

### Literature review

A literature review was carried out of peer-reviewed literature published up to March 2021, limited to English language. Studies conducted in languages other than English were excluded from the review. The review was conducted following the PRISMA (Preferred Reporting Items for Systematic Reviews and Meta-Analyses) guidelines by applying PRISMA NMA Checklist.

Articles were searched by consulting the main scientific databases on the web such as PubMed, Google Scholar, Medline, EMBASE and the Cochrane library using specific keyword pairs such as retraction pocket (OR Tympanic atelectasis OR tympanic retraction OR atelectatic otitis) AND management (OR treatment OR surgery).

The studies were selected by applying the following inclusion criteria:

1. Patients features: patients affected by RP or TM atelectasis in both symptomatic and asymptomatic forms regardless age, sex and ethnicity.
2. Type of treatment: cartilage tympanoplasty (CT) with or without mastoidectomy.
3. Outcomes: RP features (RP resolution, stabilization or progression), ABG features (improvement, stabilization or worsening), complications (TM perforation, otorrhea, need for re-intervention).
4. Studies features: randomized controlled trials (RCT), nonrandomized controlled trials (NRCT), cohort studies and case–control studies were included.

Studies were excluded if they included groups of patients with cholesteatoma, chronic otitis media with perforation or patients undergoing surgical treatments other than tympanoplasty with or without mastoidectomy. Case reports were not considered in this review. The review was carried out by two principal investigators and any decision regarding inclusion or exclusion of the studies was made after discussion and consultation with a third author.

The results of the studies were then combined, integrated and analyzed.

## Results

### Prospective study

This prospective study included 51 patients, 24 males and 27 females, affected by tympanic membrane RP. The patients had a mean age of 31.4 years (range 8–66).

In our study group all patients reported varying degrees of hearing loss which was subsequently confirmed by audiometric examination. In addition, intermittent otorrhea was reported by 11 patients.

In 34 cases, ETD was detected in tubal function tests. The evaluation of the functionality was carried out by means of a tympanometric test which made it possible to detect an average pressure lower than 50daPa in the middle ear. ETD data was also supported by the results obtained from tubal opening tests using the Valsalva and Toynbee maneuvers.

None of the patients reported previous procedures on Eustachian tube (e.g. Eustachian tube balloon dilatation) in their clinical history instead 24 patients reported previous ventilation tube placement with no benefit in symptomatology and/or progression of tympanic membrane retraction. The 11 patients who reported otorrhea had instead undergone previous medical therapy with steroids and/or nasal decongestants without any lasting benefit. The fibroenadoscopic evaluation of the nose, paranasal sinuses and nasopharynx, on the other hand, did not reveal any pathology affecting the anatomical regions explored.

Baseline characteristics of the patients involved in our study are summarized in Table 1.

**Table 1:** Baseline characteristics of the patients

	Group 1 (G1)		Group 2 (G2)		*p*	Series	
Mean age	31.9 (10–66)		31.2(8–62)		> 0.5	31.4 (8–66) years	
M/F ratio	11/13		13/14		> 0.7	24/27	
Subjective tinnitus	7 (29.2%)		6 (22.2%)		> 0.7	13 (25.5%)	
RP grade	III	IV	III	IV	> 0.7	III	IV
	11	13	13	14		24	27
RP site							
Pars tensa	15 (62.5%)		17 (62.9%)		> 0.7		32 (62.7%)
Pars flaccida	9 (37.5%)		10 (37.1%)				19 (37.3%)
Ossicular chain interruption (incus-stapes joint)	14 (58.4%)		13 (48.2%)		> 0.7		27 (52.3%)
Air–bone gap	23.8 ± 8.1 dB HL		21.3 ± 4.7 db HL		> 0.7		22.7 ± 7.2 dB HL
Otorrhea	5 (20.9%)		6 (22.3%)		> 0.5		11 (21.6%)
Reconstruction technique							
Fascia + cartilage	17 (70.8%)		17 (62.9%)		> 0.9		34 (66.7%)
Fascia	9 (29.2%)		8 (37.1%)		> 0.7		17 (33.3%)

In 32 cases RP involved the pars tensa. Pars flaccida retraction was detected in 19 cases with lateral attic wall erosion in 13 cases. Retraction of the posterior quadrants with adhesion to the promontory occurred in 31 cases. Ossicular chain interruption was detected intraoperatively in 27 cases. Anterior RP occurred only in three patients.

A higher number of cases with ear canal erosion and a lower ABG was found among patients with an RP of the pars flaccida with respect to individuals with an RP of the pars tensa (*p* < 0.05).

Clinical retraction pockets characteristics are summarized in Table 2.

**Table 2:** Clinical retraction pockets characteristics

RP site	Pars tensa	Pars flaccida	*p*
	32	19	
Scutum erosion	2	15	< 0.05
Ossicular chain interruption (incus-stapes joint)	20	7	> 0.5
ABG	28.9 ± 4.8 dB HL	14.7 ± 3.6 dB HL	< 0.05
Otorrhea episodes	7	4	> 0.5

The entire study group had a mean preoperative ABG of 22.7 dB HL. A greater ABG was detected in patients with ossicular chain involvement (32.1 dB HL vs 12.7 dB HL).

Twenty-four patients underwent tympanoplasty with mastoidectomy (G1), while 27 patients underwent only tympanoplasty only (G2). Temporalis muscle fascia graft reinforced with tragal cartilage was used for the tympanic membrane reconstruction. In the remaining 17 cases reconstruction was performed with fascia alone.

**Fig. 1:**
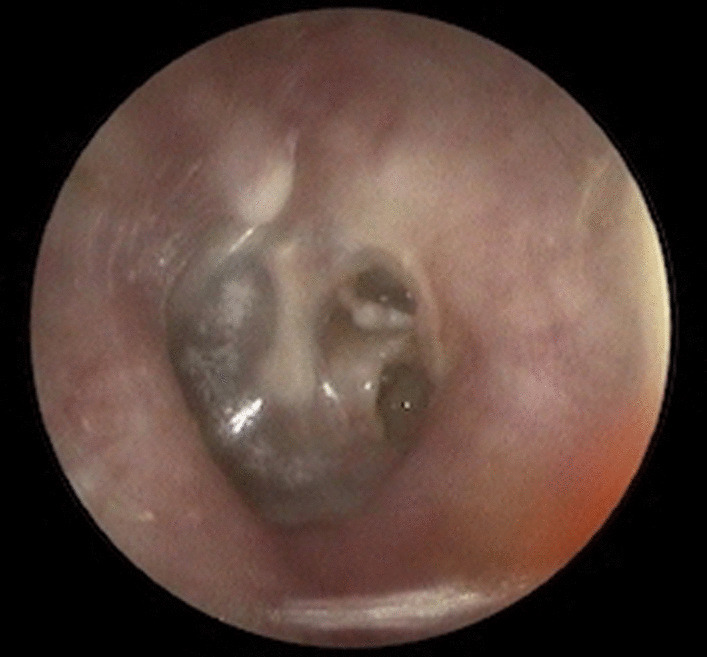
Preoperative left otoscopy, grade III RP with "not-visible fundus" and ossicular chain erosion

**Fig. 2:**
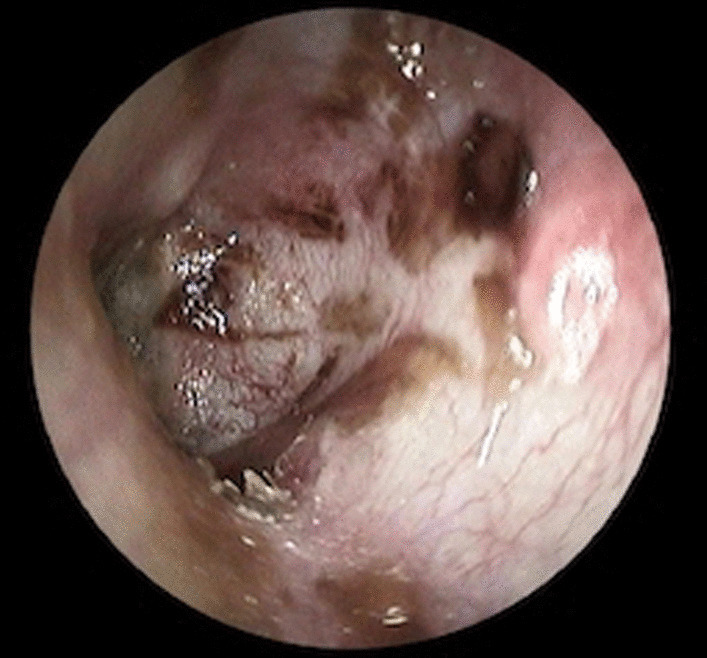
Postoperative left otoscopy, grade III RP with "not-visible fundus" and ossicular chain erosion managed with RP excision, reconstruction with temporalis muscle fascia reinforced with tragal cartilage and reshaped autologous incus

Twenty-seven patients underwent ossiculoplasty: in 14 cases it was done with autologous incus reshaped and replaced or autologous cartilage graft, while in 13 cases, a titanium partial ossicular replacement prosthesis (*PORP)* was used. None of the cases developed postoperative complications. Figures 1 and 2 show, respectively, pre- and postoperative otoscopy of a patient affected by grade III RP with “not-visible fundus” and ossicular chain erosion managed with RP excision, reconstruction with temporalis muscle fascia reinforced with tragal cartilage and reshaped autologous incus (Figs. 1 and 2).

The mean follow-up lasted about 36 months (range 12–48 months).

Average ABG decreased from 22.7 to 5.2 dB HL, the mean improvement was 13.4 dB HL (range from 5 to 35 dB HL) (Table 3).

**Table 3:** Hearing outcomes after surgery

	G1 + G2	G1	G2	*p* value
*N* of Cases	51	24	27	
Preoperative ABG	22.7 ± 7.2 dB HL	23.8 ± 8.1 dB HL	21.3 ± 4.7 dB HL	> 0.5
Postoperative ABG	5.2 ± 2.1 dB HL	7.1 ± 2.7 dB HL	5.1 ± 2.2 dB HL	< 0.05
Average ABG improvement	13.4 ± 2.3 dB HL	13.2 ± 2.9 dB HL	12.4 ± 3.4 dB HL	> 0.5
RP recurrence	4	2	2	> 0.5

The mean postoperative ABG was 7.1 and 5.1 dB HL in G1 group and G2 group, respectively, with a mean ABG improvement of 13.2 dB HL (G1) and 12.4 dB HL (G2). Patients undergone to ossicular chain reconstruction had a mean postoperative ABG of 12.7 dB HL and an average improvement of 17.2 dB HL (Table 4).

**Table 4:** Post-operative hearing outcames

	Interrupted ossicular chain	Normal ossicular chain	*p* value
*N* of cases	27	24	
Preoperative ABG	32.1 ± 5.1 dB HL	12.4 dB ± 3.1 dB HL	< 0.05
Postoperative ABG	12.7 ± 3.3 dB HL	0.5 ± 1.1 dB HL	< 0.05
Average improvement	17.2 ± 2.7 dB HL	9.3 ± 3.3 dB HL	< 0.05

An ABG improvement was observed in the 59.7% of the G1 group and in the 63.2% of the G2 group, respectively (*p* > 0.5). Recurrence of an asymptomatic grade II RP was detected in four other patients, two subjects belonging to the G1 group and two cases in the G2 group (*p* > 0.05). During the follow-up only one case from the G1 group developed a progression of the RP into cholesteatoma after 48 months. No neo-tympanum perforation was found.

### Literature review

The review yielded 2258 articles. A first screening allowed us to eliminate 1415 duplicates and, therefore, to consider only the remaining 843 articles. Some 794 articles were excluded based on title/abstract screening allowing us to select 49 articles for full-text screening. Then, the application of inclusion and exclusion criteria allowed us to select only 1 paper [11] for inclusion in the review. The exclusion of the remaining 48 papers was caused in most cases by study designs represented by case reports, by study groups including patients with cholesteatoma or chronic otitis media with perforation or by surgical treatments other than tympanoplasty with or without mastoidectomy. Studies with poor clinical data reported were also excluded.

## Discussion

RP is frequently reported in clinical ENT practice and different management strategies may be performed to treat it. Even if RP could be asymptomatic for a long time, it may develop severe complications as cholesteatoma with bone erosion after several years (with otorrhea, hearing loss, tinnitus and earache) [12, 13]. Although there is no common approach to the treatment of tympanic retraction pockets among ENTs, the literature is rich of studies carried out on this class of patients. For grade III and IV retraction pockets, however, surgery seems to be the most effective treatment [7]; in fact, different authors reported an absence of recurrence in the 67–74% of patients who undergo tympanoplasty [14–17]. Among surgical procedures, few studies investigated the efficacy of mastoidectomy in the management of RP. The rationale for mastoidectomy may consist in increasing the volume of the ME to re-establish the pressure balance with the external environment, since the pressure imbalance between these two systems is the main pathogenetic mechanism of tympanic membrane atelectasis [18].

However, in addition to presenting intrinsic risks such as damage to the facial nerve, middle ear surgery could lead to failure: in fact, in a percentage of cases, a lack of physiological re-epithelialization of the mastoid cavity can cause recurrence of the retraction pocket with failure to improve the hearing threshold.

To assess whether a mastoidectomy is needed, we conducted a prospective study on 51 consecutive patients evaluating the difference in both anatomical and functional results based on the surgical technique applied: tympanoplasty vs tympanoplasty with mastoidectomy.

In our study sample we did not notice a significant statistical difference in terms of ABG improvement between the two groups. No significant difference in terms of recurrent RP, neo-tympanum perforation and cholesteatoma was also found between G1 and G2 groups suggesting no advantage in performing mastoidectomy.

The only article retrieved from the literature review performed was the one by Ozbek et al., which evaluated whether there were differences, both in terms of anatomical and functional outcomes, in patients undergoing exclusive cartilage tympanoplasty performed with the palisade technique (27 cases) or by applying a concomitant mastoidectomy (29 cases) [11]. Cases were evaluated up to a maximum follow-up of 68 months.

Post-operative otomicroscopy revealed normal healing of the tympanic membranes in 91% of the ears. A mild or a moderate RP recurrence was instead detected in nine (16%) and five (8%) patients, respectively. Adverse events with development of tympanic membrane perforation were reported in five cases; all of them underwent revision surgery. Functional results with a reduction of ABG below 20 dB were achieved in 71% of cases. Starting from an ABG value of 28.4 ± 5.8 db HL for the whole case series, a postoperative ABG of 16.9 ± 6.7 dB was achieved regardless of the type of surgery performed with an average improvement of 11.5 dB HL (*p* < 0.001). As for the post-operative ABG results achieved by patients undergoing or not undergoing mastoidectomy, they were 11.8 and 11.2 dB HL, respectively (*p* > 0.05). The detection of these results allowed the authors to note that mastoidectomy does not significantly improve both anatomical and functional results. Same conclusions were reached within our study: only a minimal difference was found in the functional results and these revealed a slight superior improvement in ABG in the G1 group, however, without any statistical significance (*p* > 0.05). While the literature is poor in studies concerning the use of mastoidectomy in patients affected exclusively by RP, numerous investigations were carried about the role of mastoidectomy in the treatment of otitis with cholesteatoma or tympanic perforation.

According to a review performed in 2016 including also studies involving patients with otitis media with perforation, Trinidade et al. concluded that there are no differences in terms of TM healing rate and ABG improvement among patients undergoing tympanoplasty only and patients undergoing tympanoplasty with mastoidectomy [19]. Similar results were reported in two case–control studies by McGrew et al. and Albu et al. [20, 21]. In their case series, Agrawal and Bhargava took instead into consideration a group of patients suffering from chronic suppurative otitis media, also in this case the results allowed them to conclude that mastoidectomy does not improve both anatomical and audiological outcomes [22].

In a study by Avraham et al. the results obtained on 111 ears of patients affected by TM retractions of different degrees were evaluated. Twenty-seven of these patients were treated with tympanoplasty only, 84 were treated with tympanoplasty with mastoidectomy. No short-term differences were detected between the two groups, but a better ventilation of the middle ear was detected after 4 years in all those patients who did not undergo to a mastoidectomy [23].

In a study including 95 cases, Boone et al. evaluated the role of mastoidectomy in revision surgery using a cartilage–perichondrium graft applying the palisade technique. In addition, in this case no statistical differences were detected between the groups undergoing or not undergoing mastoidectomy. They concluded that probably, the presence of a rigid structure, such as cartilage, made it possible to better resist the pressure changes of the middle ear avoiding the recurrence of the TM retraction [24].

Based on this analysis it is possible to conclude that in the treatment of simple RPs, mastoidectomy should not be added to the standard surgical technique but should only be applied in case of high suspicion of cholesteatomatous otitis media, chronic otitis media or in the case of recurring RPs. Furthermore, the indication to perform mastoidectomy in patients who report tinnitus or hearing threshold deficit as their main problems, should be carefully evaluated, since mastoidectomy does not allow to resolve these symptoms which are mainly dependent on the conditions of the ossicular chain [25].

## Conclusions

Even if our data are limited by the relatively small sample size of the study, they support the existing literature suggesting no evident benefit of performing mastoidectomy in the treatment of RP. In fact, nor better ABG neither lower rate of recurrences was recognized among those patients who underwent tympanoplasty with mastoidectomy. A larger surgical series associated with a long-term follow-up are needed to confirm our conclusions with greater certainty.
